# The Potential of Pulmonary Vasodilators as a Bridging Therapy for Lung Transplantation: Two Cases of Interstitial Pneumonia With Pulmonary Hypertension During the Waiting Period for Lung Transplantation

**DOI:** 10.1002/rcr2.70136

**Published:** 2025-03-17

**Authors:** Yuichi Nagata, Tetsutaro Nagaoka, Yuriko Terayama, Yoshifumi Suzuki, Takashi Yoshida, Sachiko Kuriyama, Satomi Shiota, Masaaki Sato, Kazuhisa Takahashi

**Affiliations:** ^1^ Department of Respiratory Medicine Juntendo University School of Medicine Tokyo Japan; ^2^ Department of Thoracic Surgery The University of Tokyo, Graduate School of Medicine Tokyo Japan

**Keywords:** interstitial pneumonia, lung transplant, pulmonary hypertension, pulmonary vasodilator

## Abstract

Pulmonary hypertension (PH) associated with interstitial pneumonia (IP) has a poor prognosis, and lung transplantation (LT) is the only effective treatment option for severe cases. However, the waiting period for LT often exceeds the median survival time in these patients, and the effectiveness of pulmonary vasodilators during this period is not well established. In Case 1, a patient with scleroderma‐related IP and PH was treated with sildenafil, which resulted in improved PH and successful single‐lung transplantation. In Case 2, a patient with rheumatoid arthritis‐related IP and PH was treated with tadalafil and macitentan, leading to successful single‐lung transplantation. These cases suggest that pulmonary vasodilators may be valuable bridging therapies for patients with PH associated with IP who are awaiting LT.

## Graphical abstract

This report presents two cases of pulmonary hypertension (PH) associated with interstitial pneumonia (IP) that were successfully managed with pulmonary vasodilators while awaiting lung transplantation (LT), which ultimately led to successful transplantation.

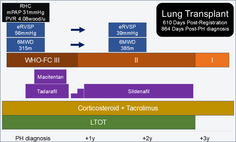

## 1 Introduction

Pulmonary hypertension (PH) associated with interstitial pneumonia (IP) has a poor prognosis. Lung transplantation (LT) is the only viable treatment option for severe cases. However, the waiting period for LT often exceeds the median survival time of these patients. Although pulmonary vasodilators have shown efficacy in improving the prognosis of PH alone, their effectiveness in patients with PH associated with IP remains unclear, and less is known about their impact on patients awaiting LT. This report presents two cases of PH associated with IP that were successfully managed with pulmonary vasodilators while awaiting LT, which ultimately led to successful transplantation.

## 2 Case Report

### 2.1 Case 1

A 41‐year‐old man was diagnosed with scleroderma‐related IP 10 years prior to LT. His condition worsened over time, and he was treated with corticosteroids, tacrolimus, and intravenous immunoglobulin, which resulted in improvement. Eight years before LT, PH was suspected based on echocardiographic findings, and long‐term oxygen therapy (LTOT) was initiated.

Two years before the LT, the patient experienced worsening dyspnoea, which led to the suspicion of PH exacerbation. The patient underwent right heart catheterisation (RHC). The results showed a six‐minute walk test (6 MWT) distance of 316 m, World Health Organisation Functional Class (WHO‐FC) III. His oxygen saturation (SpO_2_) was 97% on 3 L/min of oxygen via nasal cannula.

The patient had a history of colon cancer resection and colostomy. He had a 20‐cigarette‐per‐day smoking habit, which he quit upon diagnosis of IP. Computed tomography (CT) revealed a usual interstitial pneumonia (UIP) pattern in both lungs. A comparison of CT scans taken 1 year and 5 years ago showed no significant changes. Additionally, there was no decline in the vital capacity over time, although the diffusing capacity of the lungs decreased.

**FIGURE 1:**
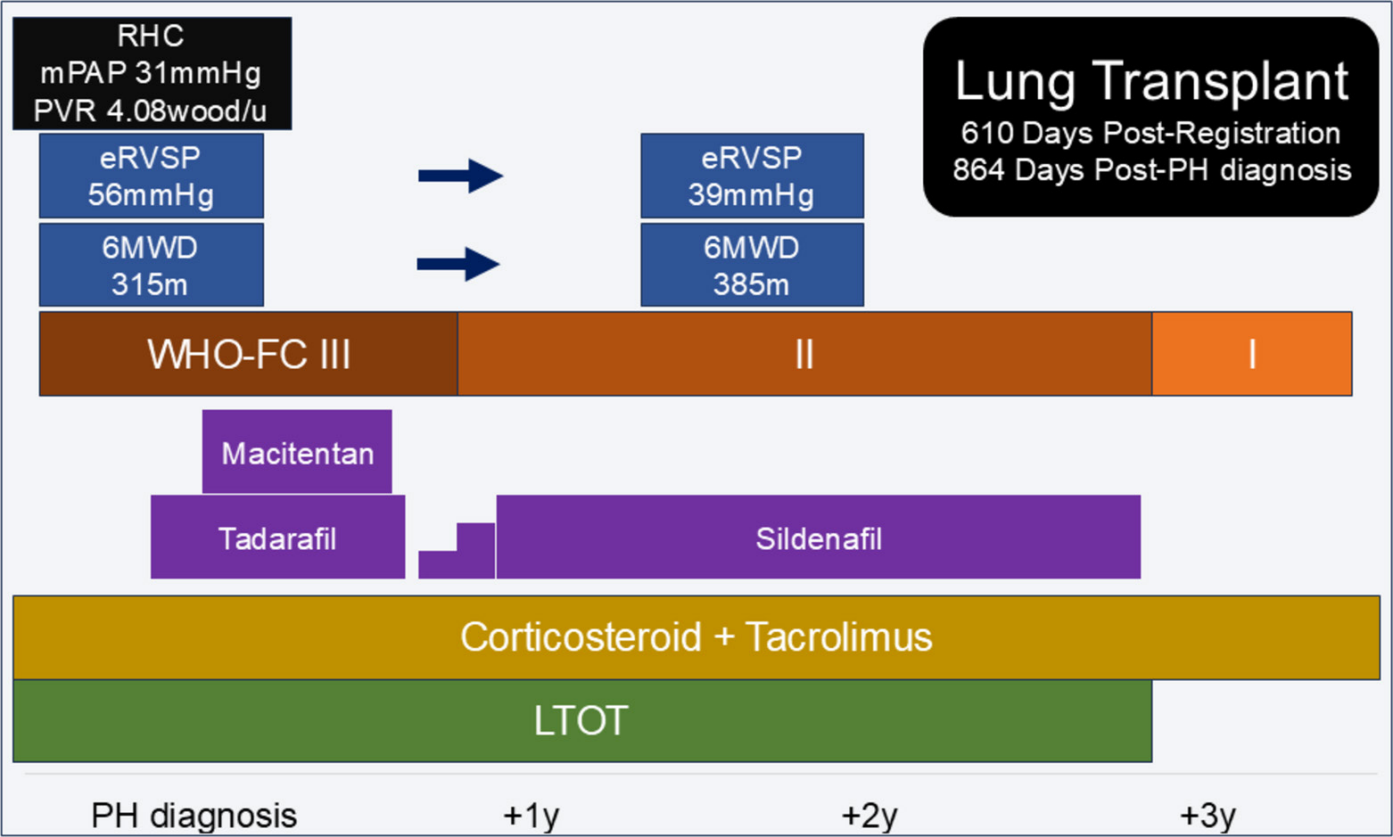
Timeline of Case 1, depicting major therapeutic interventions and changes in examination results.

The RHC results indicated a mean pulmonary arterial pressure (mPAP) of 31 mmHg, a pulmonary capillary wedge pressure (PCWP) of 12 mmHg, and a pulmonary vascular resistance (PVR) of 4.08 Wood units. Based on these findings, the patient was diagnosed with PH with only a slight worsening of the IP. He was treated with pulmonary vasodilators and listed for LT. Initially, tadalafil was administered, and macitentan was added. However, owing to persistent headaches unresponsive to NSAIDs, macitentan was discontinued, and tadalafil was switched to sildenafil (Figure 1).

After 6 months of vasodilator treatment, improvements were noted in the estimated right ventricular systolic pressure (eRVSP) (from 56 to 39 mmHg), the WHO‐FC (from class III to class II), and the 6 MWT distance (from 315 to 385 m). After confirming a stable PH with sildenafil, treatment with nintedanib for interstitial pneumonia was initiated.

The patient successfully underwent a single left lung transplantation 864 days after PH diagnosis. Currently, he no longer requires LTOT, and pulmonary vasodilators were discontinued post‐LT owing to the improvement in PH.

### 2.2 Case 2

A 39‐year‐old woman was diagnosed with rheumatoid arthritis‐related IP 7 years prior to LT. The patient was treated with corticosteroids and methotrexate. Four years before LT, she was hospitalised for right heart failure and was diagnosed with PH, with a mPAP of 27 mmHg. She was treated with sildenafil, LTOT, and non‐invasive positive‐pressure ventilation (NPPV).

Two years before LT, she was hospitalised again due to worsening dyspnoea and was treated with diuretics for right‐sided heart failure. She subsequently underwent right heart catheterisation after her condition was stabilised. Her functional status was classified as WHO‐FC III. Her SpO_2_ was 95% on 5 L/min of oxygen via nasal cannula.

The patient's family history was significant for IP in her brother and uncle and for lung cancer in her mother and cousin. She had a history of smoking 20 cigarettes per day and quit upon her diagnosis. CT revealed a non‐specific interstitial pneumonia (NSIP) pattern in both lungs. One year prior, CT revealed advanced IP and pulmonary artery dilatation.

**FIGURE 2:**
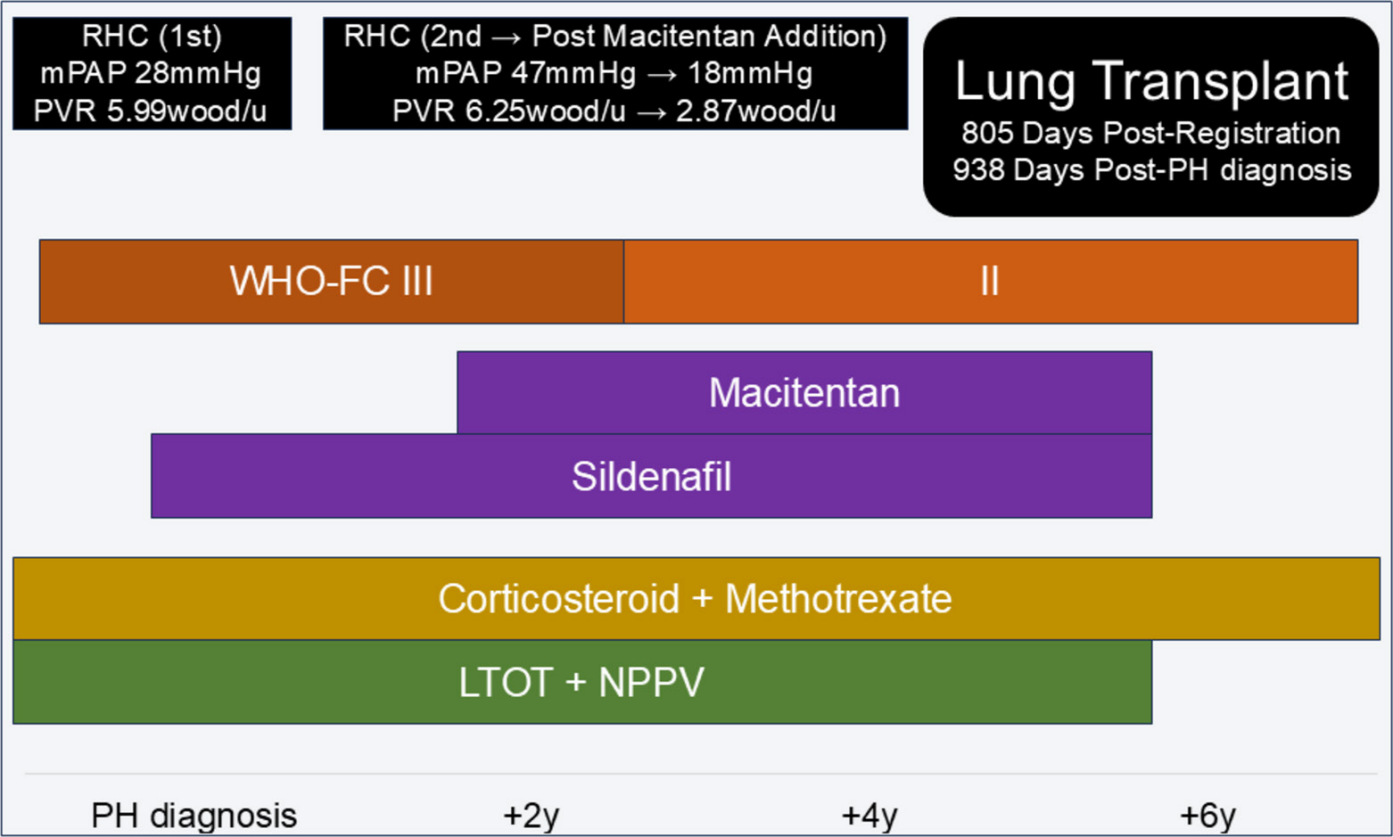
Timeline of Case 2, illustrating major therapeutic interventions and changes in examination results.

The RHC results indicated a mPAP of 47 mmHg, a PCWP of 11 mmHg, and a PVR of 6.25 Wood units. Owing to the severity of her PH, macitentan was added to her treatment regimen. Two weeks later, her mPAP improved to 18 mmHg. The patient continued vasodilator therapy while on LTOT and NPPV, resulting in a stable respiratory status (Figure 2).

The patient successfully underwent a single left lung transplantation 911 days after PH diagnosis. She currently uses LTOT intermittently. Pulmonary vasodilators were discontinued post‐LT due to improvement in PH.

## 3 Discussion

These two cases indicate the potential utility of pulmonary vasodilators as bridging therapy during the waiting period for LT. The efficacy of selective pulmonary vasodilators for IP‐PH has not been demonstrated, and aggressive treatment with these drugs is not recommended as per current guidelines [1]. However, there may be some advantages to administering pulmonary vasodilators for patients with IP‐PH before transplantation.

First, the number of patients who can undergo LT may increase. The median overall survival of patients with idiopathic pulmonary fibrosis complicated by PH (mPAP > 20 mmHg) is significantly shorter than that of those without PH [2], and the survival period is often shorter than the average waiting period for a LT in Japan, which is approximately three years [3]. The prognosis of IP with severe PH that responds to pulmonary vasodilator treatment is significantly extended [4]. Therefore, the number of patients who can undergo LT may increase among those who respond to vasodilative treatments. Second, the number of patients who choose to receive a single‐lung transplant may increase. The selection of the transplantation method (single or bilateral lungs) is critical. Previous reports on pulmonary fibrosis indicated no difference in the prognosis of bilateral lung transplantation based on preoperative mPAP. However, a preoperative mPAP ≥ 30 mmHg is associated with significantly worse outcomes for single lung transplantation [5], thus, it is preferable to perform bilateral lung transplant in such cases. Reducing mPAP with vasodilators may allow for the consideration of single lung transplantation as an option and lead to an increased opportunity for lung transplants for waiting patients, particularly in countries with insufficient donors, such as Japan. Third, it may improve the prognosis during the perioperative period of LT. The success rate of LT is influenced by the preoperative mPAP. Higher preoperative mPAP correlates with increased mortality post‐transplantation [6]. A potential cause is primary graft dysfunction (PGD). Elevated pulmonary vascular resistance and right ventricular dysfunction may cause the graft to experience high shear stress during reperfusion, contributing to PGD [7]. The use of vasodilators before transplantation to lower the mPAP could reduce the incidence of severe PGD and potentially improve the success rate of LT. For these reasons, treatment with a pulmonary vasodilator may have contributed to the successful outcomes of LT in the present case.

Treatment with pulmonary vasodilators in patients with IP should be performed with caution, as this may induce hypoxemia. Phosphodiesterase 5 inhibitors (PDE‐5i) have the effect of increasing blood flow to healthy lung areas; thus, they are less likely to cause hypoxemia due to ventilation‐perfusion mismatching [8]. While PDE‐5i are often used as first‐line pulmonary vasodilators for IP‐PH, it is important to note that even PDE‐5i can sometimes worsen respiratory failure. Therefore, additional secondary drugs should be carefully considered. In Case 1, because the early addition of the second drug led to a serious adverse event, treatment with pulmonary vasodilators had to be temporarily discontinued.

Furthermore, in cases of IP‐PH, not only the management of PH but also that of IP itself is crucial. Some patients do not survive due to chronic progression or acute exacerbation of IP while waiting for lung transplantation. The appropriate introduction of antifibrotic agents may contribute to successful lung transplantation. In Case 1, we believe that nintedanib was administered at an appropriate time before transplantation.

Recently, inhaled treprostinil has been reported to improve exercise tolerance and clinical course in patients with IP‐PH, and its use has become feasible in several countries [9]. Furthermore, it has also been shown that inhaled treprostinil may prevent the decline of restrictive ventilatory impairment, and it is expected to have a direct effect on IP progression. In the near future, inhaled treprostinil will likely be selected as the first‐line pulmonary vasodilator for patients with IP‐PH waiting for transplantation.

In conclusion, pulmonary vasodilators demonstrated good tolerability and improved haemodynamics, even in patients with severe respiratory failure. In patients with severe IP and PH, pulmonary vasodilators, including inhaled treprostinil, may be useful bridging therapies before LT.

## Author Contributions

Y.N., Y.T., Y.S., T.Y., S.K., and K.T. were the attending physicians who treated the patient on admission. T.N. and S.S. were outpatient physicians. M.S. was a transplant surgeon. Y.N., T.N., and K.T. drafted the manuscript. Y.N. submitted the final manuscript. All authors have read and approved the final manuscript for submission.

## Ethics Statement

The authors declare that appropriate written informed consent was obtained for the publication of this manuscript and accompanying images.

## Conflicts of Interest

Kazuhisa Takahashi is an Editorial Board member of Respirology Case Reports and a co‐author of this article. He was excluded from all editorial decision‐making related to the acceptance of this article for publication. The other authors declare no conflicts of interest.

## Data Availability

Data sharing is not applicable to this article as no new data were created or analyzed in this study.
